# Antibody-mediated disruption of the mechanics of CS20 fimbriae of enterotoxigenic *Escherichia coli*

**DOI:** 10.1038/srep13678

**Published:** 2015-09-28

**Authors:** Bhupender Singh, Narges Mortezaei, Bernt Eric Uhlin, Stephen J. Savarino, Esther Bullitt, Magnus Andersson

**Affiliations:** 1 Department of Physics, Umeå University, SE-901 87 Umeå, Sweden; 2 The Laboratory for Molecular Infection Medicine Sweden (MIMS) and Department of Molecular Biology, Umeå University, SE-901 87 Umeå, Sweden; 3 Enteric Diseases Department, Infectious Diseases Directorate, Naval Medical Research Center, Silver Spring, MD 20910, USA; 4 Department of Pediatrics, Uniformed Services University of the Health Sciences, Bethesda, MD 20814, USA; 5 Department of Physiology and Biophysics, Boston University School of Medicine, Boston, MA 02118, USA

## Abstract

Preventive vaccines against enterotoxigenic *Escherichia coli* (ETEC) are being developed, many of which target common fimbrial colonization factors as the major constituent, based on empirical evidence that these function as protective antigens. Particularly, passive oral administration of ETEC anti-fimbrial antibodies prevent ETEC diarrhea. Little is, however, known regarding the specific mechanisms by which intestinal antibodies against ETEC fimbriae function to prevent disease. Using coli surface antigen 20 (CS20) fimbriae as a model ETEC colonization factor, we show using force spectroscopy that anti-fimbrial antibodies diminish fimbrial elasticity by inhibiting their natural capacity to unwind and rewind. In the presence of anti-CS20 antibodies the force required to unwind a single fimbria was increased several-fold and the extension length was shortened several-fold. Similar measurements in the presence of anti-CS20 Fab fragments did not show any effect, indicating that bivalent antibody binding is required to reduce fimbrial elasticity. Based on these findings, we propose a model for an *in-vivo* mechanism whereby antibody-mediated disruption of the biomechanical properties of CS20 fimbriae impedes sustained adhesion of ETEC to the intestinal mucosal surface. Further elucidation of the role played by intestinal antibodies in mechanical disruption of fimbrial function may provide insights relevant to ETEC vaccine development.

Enterotoxigenic *Escherichia coli* (ETEC) are a major bacterial cause of diarrhea in children in resource-limited regions and in travelers^1^,^2^. Well-recognized pathogenic features of ETEC are adhesive fimbriae that promote intestinal adherence and colonization, and enterotoxins that induce enterocytes to secrete electrolytes and fluid^3^. Among the ETEC fimbrial colonization factors that have been described in association with human disease are coli surface (CS) antigen 12 (CS12), CS18, CS20, and CS26^4^,^5^,^6^,^7^. These fimbriae are genetically related and along with the pig-specific ETEC fimbriae 987P are grouped as Class 1b fimbriae^4^,^6^. Extrapolating from detailed studies of 987P, class 1b fimbriae are composed of a major, immunodominant subunit that forms the helical stalk, and two minor subunits one of which functions as a tip-localized adhesin^8^. The identification of new members of Class1b fimbriae from ETEC isolated from Egyptian children with severe diarrhea suggests a greater importance than previously appreciated^7^. First reported from India, CS20 has been detected in 6–7% of collections of ETEC disease isolates from India and Egypt^7^,^9^. Using CS20 as a model Class 1b ETEC fimbriae, we have recently elucidated its ultrastructural features at high resolution and demonstrated its pliability in that the helical rod is capable of unwinding and rewinding under forces normally encountered in the intestinal tract^10^.

Over the past decade, efforts have expanded to develop an effective vaccine against ETEC diarrhea to stem its resultant morbidity and mortality in young children and morbidity in travelers^11^,^12^,^13^. Fimbrial colonization factors or their derivatives are a central component of most vaccines in clinical development^14^,^15^,^16^. Among the various lines of evidence indicating the suitability of fimbriae as ETEC vaccine targets, passive oral prophylaxis with hyperimmune anti-fimbrial colostral antibodies conferred protection to volunteers who were challenged with fimbriae-homologous ETEC^17^. The exact mechanisms by which antibodies prevent ETEC diarrhea have not been explicitly defined, though inhibition of epithelial attachment, bacterial aggregation, entrapment in mucus and enhanced bacterial clearance are rational possibilities^18^.

In recent work, another mechanism has been proposed by which antibodies might impair bacterial infection on mucosal surfaces. Results from *in-vitro* experiments show that shaft-specific antibodies can inhibit the unwinding and rewinding of P-fimbriae, a well-established virulence factor of uropathogenic *E. coli* (UPEC)^19^. This is the only instance reported to date in which antibodies cause biomechanical disruption of fimbrial elasticity, and it is unclear whether other classes of fimbriae would be similarly affected. Because of their central role in pathogenesis of intestinal rather than urinary tract disease, adhesive fimbriae of ETEC provide a distinct model for exploring the generalizability of this hypothesis. Moreover, ETEC fimbriae, including CS20, have been shown to feature biomechanical and structural properties that are altogether distinct from that of P-fimbriae^10^,^20^,^21^. In the present work, we tested the effect of fimbriae-specific antibodies to CS20 under controlled conditions. Using optical tweezers force spectroscopy, we studied the biomechanics of individual CS20 fimbria^22^,^23^. We observed significant reduction in the unwinding and rewinding responses of individual CS20 fimbriae exposed to antibodies. Since it has been proposed that fimbrial unwinding and rewinding may play an important role modulating bacterial adhesion^18^,^24^, our findings have implications for the development and refinement of ETEC vaccines.

## Results

### Mechanics and dynamics of CS20 fimbriae

**Figure 1:**
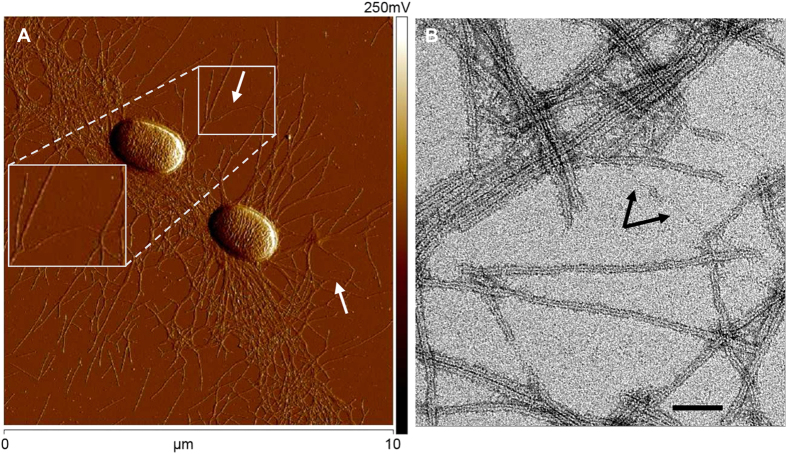
CS20 fimbriae. (**A**) Atomic Force Microscopy (AFM) image showing two bacterial cells (strain WS7179A-2/pRA101) expressing CS20 fimbriae under normal growth. Fimbriae are rarely found in an unwound state (white arrows). The insert shows a zoomed-in view of a fimbria that has made a dynamic change into a fibrillar structure. Scale bar is 10 μm. (**B**) Transmission electron microscopy image of negatively stained, purified CS20 fimbriae. Arrows show unwound regions of a fimbria, separated by a short helical (wound) region. Scale bar is 50 nm.

**Figure 2:**
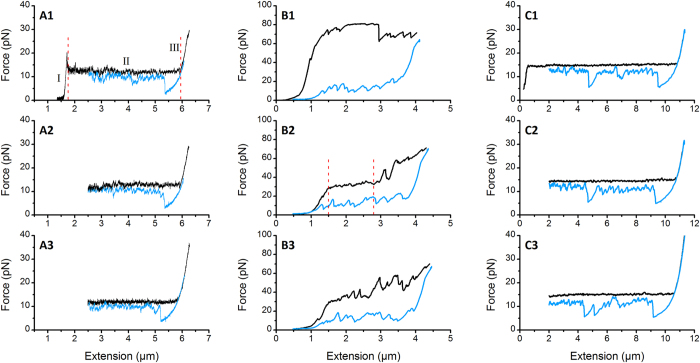
Force spectroscopy experiments of a single CS20 fimbria. The black and blue curves represent unwinding and rewinding, respectively, of a fimbria close to steady-state. Panels A1-A3 show typical consecutive force measurements of unaccompanied CS20 fimbria. The unwinding force initially shows a linear increase in region (I) that originates from elastic elongation of the fimbria. A constant force plateau in region (II) represents sequential opening of the quaternary structure of fimbria, and region (III) shows tensile elastic stretching of the subunits. Panels B1-B3 show three consecutive measurements of a single CS20 fimbria in the presence of 2.8 μg/ml anti-CS20 antibodies. Panel B1 shows the first phase of unwinding and rewinding of the fimbria being influenced by antibodies: during unwinding the force increases significantly (~80 pN) indicating that higher force is required to open helical layers of the shaft that have been clamped by antibodies. During unwinding there are peaks in the force; these peaks represent detachment of one arm of the antibodies that eases subsequent opening of the helical layers in the second phase of extension (panel B2). The force level reduces considerably during rewinding of the fimbria showing that it cannot regain its helical form. The exception is the presence of force-peaks that show small sections of the shaft have been rewound. In panel B2 the same fimbria is unwound and rewound for the second time. A constant force plateau in the beginning of the curve (between dashed vertical lines) represents a sequential unwinding of the shaft. Panel B3 shows the third extension-contraction phase where the unwinding curve includes only a very small force plateau. Beginning at ~2 μm extension, the non-linear force increase shows that layers are mostly opening stochastically. The few force peaks that are observed during rewinding indicate a few formed helical layers. Panels C1-C3 demonstrate unwinding and rewinding of CS20 fimbriae in the presence of Fab fragments. An intact force plateau indicates that Fab fragments do not impair the unwinding, whereas additional force drops during rewinding show the impact of Fab fragments on rewinding of fimbriae.

Atomic force microscopy of the ETEC strain, WS7179A-2/pRA101, displayed bacterial morphology with CS20 fimbriae clearly visible around the cell, as shown in a representative micrograph obtained by AFM analysis (Fig. 1). Fimbriae are seen individually and in bundles, where the latter is an effect of assessing the images on a dry surface. The majority of fimbriae take on a helical shape (thick filaments), and occasionally unwound fimbriae were observed (narrow filaments, white arrows). Previously, we characterized the structural and mechanical properties of CS20 fimbriae and showed that CS20 can be unwound^10^. In this work we successfully reproduced the force-extension curve characteristic of CS20 fimbriae. Figure 2 (see panels A1 to A3) shows consecutive force measurements of a single CS20 fimbria under steady-state conditions (thermodynamic equilibrium) with an extension velocity of 0.05 μm/s. The extension force curves of a CS20 fimbria show three distinct unwinding regions, a pattern also observed with other classes of fimbriae^25^,^26^,^27^. In Region-I, linear increase of the force represents elastic stretching of the fimbrial shaft. In Region-II, a constant force plateau results from sequential unwinding of the shaft subunits. This region is marked by the two vertical dashed red lines in Fig. 2, panel A1. Including data (n = 58) from our previous study^10^, CS20 fimbriae unwind at a constant force of 15 ± 1 pN, n = 74. Finally, Region-III shows increasing force resulting from tensile stretching of the subunits now in an open coiled structure.

The rewinding force curve follows that of the extension curve except for a drop in force, which for the representative data occurs at 5.4 μm on the x-axis (see Fig. 2, panel A1). This drop is a consequence of rewinding a structure in a stretched, linearized form into a helical shape that is held together by layer-to-layer interactions. The fimbria needs a certain amount of slack so that the subunits participating in these interactions are in close proximity^28^. The slightly lower force during rewinding results from the fact that the rewinding cycle is not being performed in complete steady-state conditions, although this does not affect the sequential unwinding cycle.

### Modeling the CS20 fimbrial force response with Monte Carlo simulation technique

**Figure 3:**
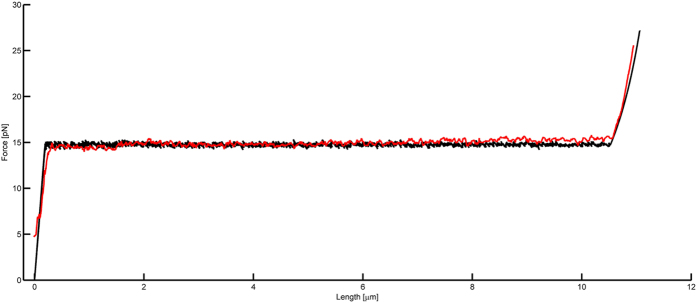
Monte Carlo simulation of a CS20. Simulated force-extension curve under steady-state of a CS20 fimbria with 1000 subunits calculated with known input parameters such as bond length, subunits per turn, and unwinding force. The red line represents experimental data of CS20 and the black line represents a Monte Carlo simulation of the model presented in Supplementary Materials. The model was optimized to fit the experimental data by minimizing the discrepancy between simulated and measured data and, as can be seen, the simulation of the model can excellently reproduce the experimental data. The model parameters are given in Table S1. The extension velocity was 0.05 μm/s in this simulation.

To further elucidate the force-extension mechanics of CS20 fimbriae, we applied a model used for describing subunit-assembled macromolecules and solved for model parameters using a Monte Carlo simulation technique^29^. The model combines Bell-Evans theory for bond kinetics in a two-state energy landscape with an elastic model in combination with a worm-like chain. The geometry of the model in this work is taken from the three-dimensional reconstruction data of CS20 fimbria^10^. We assembled the subunits in the model with 3.21 subunits per turn and used a bond length of 0.4 nm. A fit of this model (black) to force spectroscopy data (red) is shown in Fig. 3, with the corresponding model parameters presented in Table S1. We conclude that the model fits remarkably well to the experimental data (Fig. 3).

### Anti-fimbrial antibodies change the mechanics of CS20 - increasing the required extension force and impairing rewinding

**Figure 4:**
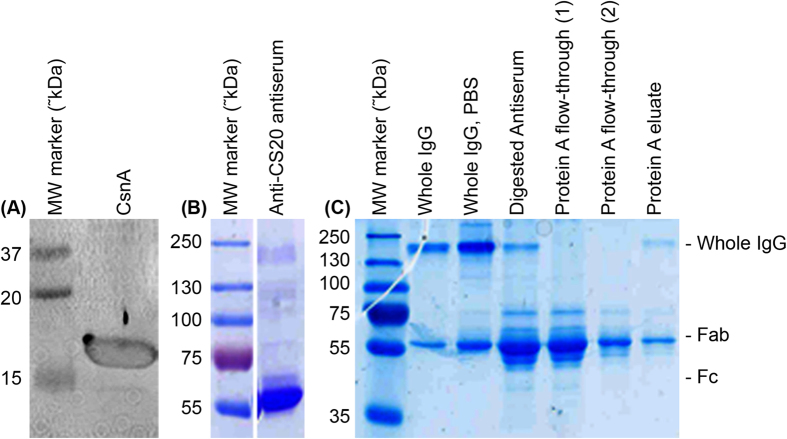
Analyzing anti-CS20 antibodies and Fab preparation. (**A**) Western blot analysis using anti-CS20 antiserum showed a specific band of approximately 17.5 kDa, the molecular weight of the CsnA structural subunit. (**B**) Anti-CS20 antiserum analyzed by non-reducing and non-boiled SDS-PAGE (12%), stained with PageBlue™, showing an IgG specific band at approximately 150 kDa and some non-specific bands. (**C**) IgG were purified using Amicon® pro Affinity Concentration Kit – Protein A (column 2). Following purification, IgG suspension buffer was exchanged to our working buffer, 1xPBS (pH 7.4) (column 3). Fab and Fc fragments were prepared by digestion of antiserum with papain. Column 4 shows digested antiserum, Protein A flow-through fractions 1 and 2, which lack any visible whole IgG, were harvested as Fab preps and were used for subsequent assays (columns 5, 6). Protein A eluate contains a mixture of undigested IgG, remaining Fab and Fc fragments (column 7). First column of each subset - A, B and C was loaded with a protein-marker indicating approximate molecular weight of protein in kDa. The figure has been cropped for clarity.

To test whether anti-fimbrial antibodies can disrupt the mechanics of CS20 fimbriae, we used purified polyclonal antibodies raised against purified CS20 fimbriae, in which the immunodominant component is the CsnA subunit that forms the fimbrial shaft. The molecular weight of purified antibodies appears close to that of immunoglobulin G (IgG) class; purification of antibodies was analyzed by means of SDS-PAGE, see Fig. 4.

Force spectroscopy experiments were done using three different concentrations of purified antibodies, 2.8, 0.28, and 0.028 μg/ml, respectively. In Fig. 2 panels B1 to B3, three consecutive unwinding and rewinding curves of a CS20 fimbria are shown in the presence of 2.8 μg/ml of anti-CS20 antibodies with panel B1 representing the first of the series. In addition, consecutive curves at the lower concentrations are presented in Figs S1 (0.28 μg/ml) and S2 (0.028 μg/ml). As can be seen in Fig. 2 (panels B1-B3), Fig. S1 and S2, the force curves were significantly changed both during unwinding and rewinding in the presence of antibodies as compared with Fig. 2 (panels A1-A3) that showed native CS20 mechanics in the absence of antibodies.

Analysis of fimbrial extension forces showed significant alterations in fimbrial biomechanics. In the presence of 2.8 μg/ml antibodies, the force required to extend CS20 was occasionally several fold higher. For example, in Fig. 2 panel B1 the extension force reaches ~80 pN (black curve) and drops to 70 pN, whereas in the subsequent measurements force peaks can be seen in the range of 40–60 pN. We suggest that the high force seen in the first measurement originates from extension of several fimbriae, which rarely was seen in the absence of antibodies. Force peaks (the force above the unwinding force) are always seen in the presence of antibodies. The force spectroscopy measurements at 100 fold lower concentration of anti-CS20 antibodies also pointed to initial unwinding that was significantly changed even though force peaks in consecutive measurements did not occur as frequently (Fig. S2).

The rewinding of fimbriae (blue curves) showed significant drops in the force level in the presence of anti-CS20 antibodies for all concentrations. For example, in Fig. 2 panels B1 to B3 the rewinding does not occur close to the unwinding force, which is the case in the absence of antibodies. The force drops even below 10 pN for almost the complete cycle.

### Extension length of CS20 fimbriae was reduced in the presence of antibodies

**Figure 5:**
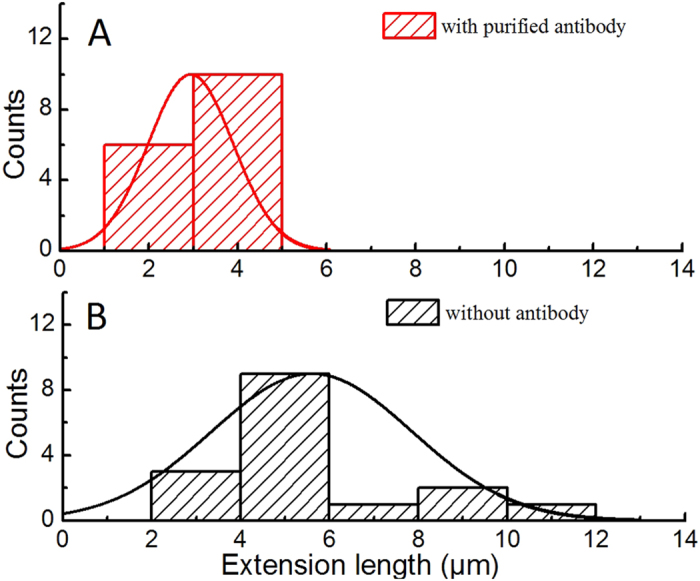
Distribution of the extension length of CS20 fimbriae in the presence or absence of antibodies. Force spectroscopy data from single fimbriae were analyzed according to extensibility. Panel A shows the distribution of unwinding lengths, mean value of 2.9 ± 0.9 μm, in the presence of anti-CS20 antibodies (2.8 μg/ml). Panel B (black) shows the distribution of the unwinding lengths, mean value of 5.6 ± 2.2 μm, in the absence of antibodies. Each distribution consists of n = 16 fimbriae. A comparison between the mean values of the two presented distributions shows that the unwinding length is strongly influenced by antibodies.

As illustrated in Fig. 2 (see panels A1-A3 and B1-B3), anti-CS20 antibodies not only altered the force required for fimbrial extension but also reduced the full extension length of fimbriae. Since experiments were performed on single fimbria, we quantified the extension lengths of several individual CS20 fimbria in the presence and absence of antibodies (*n* = 16 each; Fig. 5). We compared the lengths of the constant force plateaus, the length between two points on the curve where the force was not altered by a force peak, as indicated by the vertical red dashed lines in Fig. 2 panel A1 (~4 μm) and panel B2 (~1.3 μm). The mean extension length (constant plateau) of CS20 fimbriae in the absence and presence of antibodies (2.8 μg/ml) were 5.6 ± 2.2 and 2.9 ± 0.9 μm, respectively. These differences were statistically significant using a student t-test analysis (p < 0.05).

### Intact antibodies are required for interference with fimbrial mechanics

The specific binding of anti-CS20 antiserum to the CsnA shaft-forming subunit of CS20 was confirmed by western blot analysis (see Fig. 4A). Next, we assessed the importance of bivalent binding of anti-CS20 antibodies to the targeted epitopes. For this assay, first we prepared monovalent fragments of antigen-binding (Fab) by cleaving intact antibodies with papain, followed by assessing the force response of CS20 fimbria in the presence of monovalent anti-CS20 Fab. Using force spectroscopy measurements, three consecutive force curves of CS20 were generated in the presence of 3.0 μg/ml anti-CS20 Fab fragments (Fig. 2, panels C1-C3), in which the extension curves were comparable to those obtained in the absence of antibodies (Fig. 1, panels A1-A3), and were altogether different to those obtained in the presence of antibodies (Fig. 2, panels B1-B3), indicating that intact bivalent antibodies are crucial for effective interference with fimbrial biomechanics. However, the rewinding force curves, in the presence of anti-CS20 Fab, showed fluctuations at random locations. We expect that extension of CS20 fimbriae might expose internal epitopes to which monovalent Fab can bind and thus interfere with the rewinding process.

## Discussion

CS20 fimbriae are representative of an increasingly recognized class of fimbrial colonization factors expressed by ETEC. Only recently has attention turned to elucidation of the fine structure and biomechanical function of these and related macromolecular virulence machines^5^,^30^. CS20 are helix-like fimbriae that can unwind and rewind at a constant force thus transmitting reduced load to the adhesin when bacteria are exposed to fluid flow^10^. In this study we investigated at a single fimbria level if antibodies primarily directed against the fimbrial shaft can change the bio-mechanical properties of CS20.

A physical model describing the extension of a subunit assembled macromolecule was applied and simulated using Monte Carlo technique to reproduce the force-extension response of a CS20 fimbria. The model remarkably reproduced the response and validated that CS20 behaves similarly to that of other helix-like fimbriae, i.e. they extended under a constant force due to sequential unwinding of subunits. The best fit was found with an energy barrier of 18.0 *kT,* when using the bond opening length (5.0 nm) and the distance to the transition barrier (0.4 nm) values assessed from experiments in previous dynamic force spectroscopy experiments^10^. These values are in agreement with parameter values found for other helix-like fimbriae^21^,^29^. The mechanics of CS20 are similar to that of the closely related CFA/I fimbriae expressed by ETEC, although CFA/I unwinds at 7.5 pN which is half of the force (15 pN) required for CS20 unwinding^21^. In contrast, the P and Type 1 fimbriae commonly expressed by UPEC unwind at 28 pN and 30 pN, respectively, which is twice as high as for CS20. Comparison of the unwinding force of fimbriae expressed by UPEC and ETEC thereby opens up an interesting question as to whether the unwinding forces of adhesive fimbriae reflect some selective features of different environmental conditions.

Force curves of CS20 fimbriae in the presence and absence of purified anti-CS20 antibodies were compared. We showed that only intact bivalent antibodies changed the flexible properties of fimbriae, such that a higher force was required to unwind the shaft and the extension length under a constant force was significantly reduced. These findings are also in concordance with force spectroscopy studies showing antibody-mediated interference of P-fimbrial mechanics^19^. To investigate if reduced fimbrial elasticity was a consequence of bivalent binding of antibodies, we compared force curves assessed in the presence of monovalent Fab fragments. The data clearly indicated that bivalent binding is necessary for reduced fimbrial elasticity. Anti-CS20 Fab fragments did not affect elasticity, but they limited the capacity of CS20 fimbriae to rewind, as indicated by the increased misfolding (Fig. 2C, blue curve).

**Figure 6:**
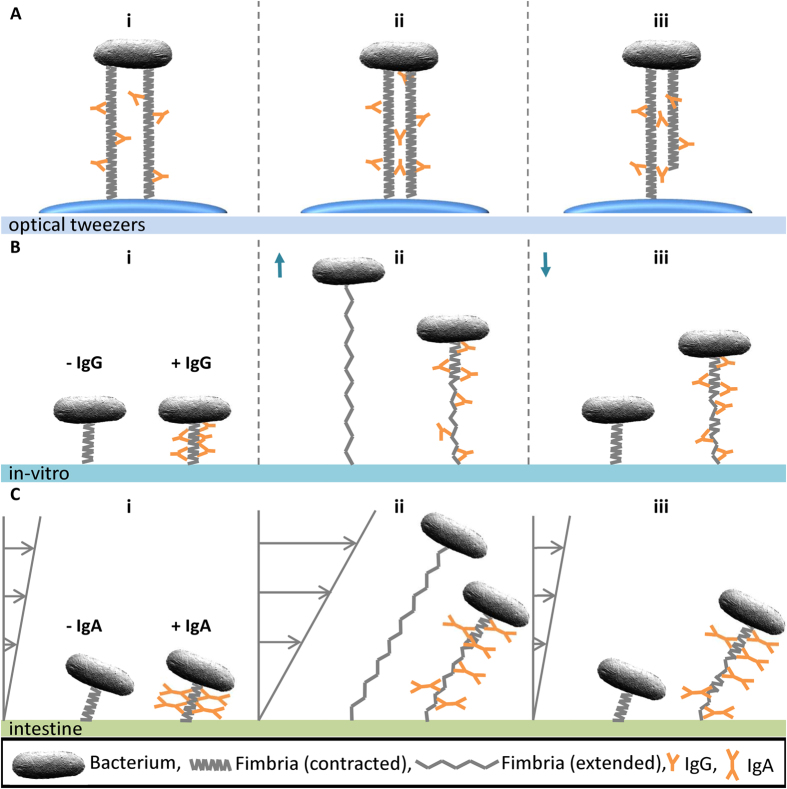
Conceptual modeling of anti-CS20 antibodies interaction with CS20 fimbriae. Panel A shows binding mode possibilities of IgG antibodies in optical tweezers force measurements, with a bacterium immobilized at the top side that is attached via fimbriae to a trapped bead (at the bottom). The illustrations show i) intra-connected antibodies locking layers of two fimbriae that are independently attached to the trapped bead (blue), ii) intra- and inter-connected antibodies, locking layers and linking two fimbriae, both of which are attached to the trapped bead due to close proximity via inter-fimbria connections, iii) intra- and inter-connected antibodies both locking layers and linking two fimbriae where only one is attached to the trapped bead. Panel B shows the *in-vitro* model (force measurement) as a time series of two bacteria attached to a surface with a fimbria in the absence (left) and in the presence (right) of antibodies. In (i) both fimbriae are shown prior to extension. In (ii) fimbriae are extended resulting in complete unwinding and partial unwinding in the absence and presence of antibodies, respectively. In (iii), after contraction the fimbria in the absence of antibodies is able to regain its initial form whereas in the presence of antibodies the fimbria cannot fully rewind. For clarity, the bead is not shown. Panel C shows a suppositional model when ETEC are attached to the intestine in the absence (left) and presence (right) of sIgA. In i) bacteria are exposed to low shear forces. In ii) the presence of high shear forces the fimbria in the absence of sIgA unwinds, reducing the load on the adhesin, whereas the fimbria exposed to sIgA only partially unwinds. In iii) subsequent low shear allows the fimbriae to regain their shape. However, in the presence of IgAs the flexibility of the fimbria is reduced and will not properly be able to damp future high fluid shear forces.

We suggest that the high unwinding force seen in our measurements, in the presence of anti-CS20 antibodies, originates from inter- and intra-fimbrial cross linking of fimbrial-shaft by bivalent antibodies leading to either simultaneous extension of several fimbriae (inter-fimbrial) or interlocking of shaft subunits (intra-fimbrial) or both, which was rarely seen in the absence of antibodies^10^. In Fig. 6, we propose conceptual models that fit our force spectroscopy data as presented in Fig. 2 (panels B1-B3). We expect that multiple fimbriae are bundled via these bivalent antibodies and either one or more of these fimbriae are attached to the trapped bead (Fig. 6A) Thus, the bivalent binding capabilities of antibodies could result in simultaneous extension of two or more fimbriae (analogous to pulling on several springs in parallel) significantly increasing the stiffness in the system (Fig. 6Ai,ii). Other scenarios represent situations where only one fimbria is attached to the trapped bead, however, inter-connected antibodies link the two fimbriae that both have attached intra-connected antibodies (Fig. 6Aiii). The *in-vitro* model presented in Fig. 6B shows how antibodies would cause tethering of layers in the shaft resulting in an increased resistance to unwinding. Antibodies would also prevent proper rewinding, implying that layer-to-layer interactions that are important for constant force response during load reduction (left cell) are compromised by the presence of antibodies (right cell).

Since secretory IgA (sIgA) plays an important role in mucosal immunity, we adapted the conceptual model to account for presumed anti-fimbrial IgA activity *in-vivo*. In Fig. 6, panel C, we illustrate how an ETEC bacterium attached via an adhesive fimbria to the host intestinal surface without (left) and with (right) bound, fimbrial-specific IgA, would appear in the presence of fluid flow. Bacterial attachment and colonization in the intestine is constantly challenged by peristaltic movements and fluid forces^31^,^32^. A bacterium can bear these shear forces by unwinding its fimbriae and thereby lowering the stress on the adhesin-receptor interaction to a level optimized for the adhesin (left cell), similar to what has been hypothesized for the Type 1 fimbriae^24^,^33^. Thus, fimbriae expressed by a bacterium that is coated by anti-shaft IgA will be unable to lower the stress on the binding mediated by a tip-localized adhesin due to the compromised extension capabilities and dampening properties (right cell). One approach for testing this hypothesis experimentally is suggested by the work of Rangel *et al.*, in which the mechanical properties of Type 1 fimbriae expressed by *E. coli* bacteria were investigated using a parallell flow chamber technique^34^.

While efforts have intensified to develop effective vaccines against ETEC diarrheal disease using preparations of immunogens based on common ETEC fimbrial colonization factors, we do not have a clear understanding of the specific mechanisms by which such a vaccine achieves protection. The findings presented herein suggest a mechanism that may contribute to this protective effect, whereby antibodies directed against the fimbriae will interfere with its biomechanical properties and thereby disrupt the finely orchestrated process by which bacteria achieve initial attachment to the intestinal epithelium under the shear stress of peristaltically driven flow. The proposed mechanism involves antibody-mediated stiffening of the fimbrial shaft, a resultant diminution in the dampening capacity of the shaft when shear forces are applied along its long axis, and ultimately increased breakage of the bond between the tip-localized fimbrial adhesin and its intestinal receptor. Further elucidation of this and other potential mechanisms will presumably help in the development and refinement of vaccines against ETEC and related bacterial pathogens.

## Material and Methods

### Bacterial strains and growth conditions

*Escherichia* coli strain WS7179A-2/pRA101 expressing CS20 fimbriae was grown overnight on CFA plates supplemented with 50 μg/ml kanamycin at 37 °C^35^. Expression of CS20 fimbriae was directly assessed by atomic force microscopy, see Fig. 1A.

### Generation of antisera against CS20 fimbriae

CS20 fimbriae were purified to near homogeneity from WS7179A-2/pRA101 using serial MgCl_2_ precipitation as described previously^36^. Rabbits were serially immunized with this fimbrial preparation by parenteral injection along with Freund’s adjuvant, and the resulting antiserum was collected by Harlan Laboratories, Inc. (Madison, WI). Specificity of anti-CS20 antiserum (R3076) was analyzed using Western blot analysis, where it recognized a 17.5 kDa band representing the CS20 major subunit CsnA (Fig. 2A).

### Antibody purification and fragments antibody binding (Fab) preparation

Polyclonal antibodies of Immunoglobulin G (IgG) isotype were purified from anti-CS20 antiserum (R3076) using Amicon® Pro Affinity Concentration Kit - Protein A, catalogue number - ACR5000PA. In brief, antiserum was allowed to interact with Protein A bound to resin. IgG, via their Fc part, were then linked to Protein A at higher pH and any other protein impurities were washed off, followed by pure IgG elution at low pH. IgG elution buffer was then exchanged with 1x PBS (pH 7.4) using 100 NMWL Amicon® Ultra-0.5 device (Fig. 2B,C – columns 2,3).

Fab fragments were prepared from anti-CS20 antiserum using Pierce Fab Preparation Kit, catalogue number – 44985. Briefly, antibodies were digested with papain which cleaves the IgG at hinge region yielding 2 Fab: 1 Fc fragments per IgG molecule. At high buffer pH, undigested antibodies and Fc fragments were linked to Protein A present in the resin column, while Protein A flow-through contained only Fab fragments. Antiserum, purified antibodies, Fab and Fc fragments were analyzed by electrophoresis under non-reducing and non-boiled conditions using 12% polyacrylamide gel containing sodium dodecyl sulfate (SDS-PAGE), stained with PageBlue™ (R0571, Fermentas), as shown in Fig. 2B,C^19^. For the ease of presentation, we labelled antiserum raised against CS20 fimbriae as anti-CS20 antiserum and purified IgG as anti-CS20 antibodies.

### Atomic force microscopy imaging

Atomic force microscopy imaging of bacterial cells was obtained using a procedure described earlier^37^. Initially cells were resuspended in 50 μl of filtered water, 10 μl of which was placed onto freshly cleaved ruby red mica (Goodfellow Cambridge Ltd., Cambridge). The cells were then incubated for 5 minutes and blotted dry before they were placed into a desiccator for a minimum of 2 h. Micrographs were collected with a Nanoscope V Multimode8 AFM equipment (Bruker software) using Bruker ScanAsyst mode with Bruker ScanAsyst-air probe oscillated at resonant frequency of 50–90 kHz.

### Sample preparation and force spectroscopy measurements

Samples were prepared as follows. CML latex beads of 9.5 μm (product no.2-10000, Interfacial Dynamics Corp.) were coated with 0.01% Poly-L-Lysine (Catalogue #P4832, Sigma-Aldrich) and immobilized on 24 × 60 mm coverslips (no.1, Knittel Glass). A chamber was made around the immobilized beads with two 5 × 15 mm strips of double-stick tape placed 5 mm apart, and covered with a 20 × 20 mm coverslip (no.1, Knittel Glass). A 4 μl mixture of pure bacterial cells with or without either antibodies, or Fabs, and 2.5 μm Surfactant-Free White Amidine Polystyrene Latex beads (product No. 3-2600, Invitrogen), in 1x PBS (pH7.4) was injected into the chamber. The ends of the chamber were sealed by vacuum grease to prevent evaporation of the suspension^19^. A sample was thereafter placed in the optical tweezers (OT) instrument to perform force spectroscopy measurements.

Detailed descriptions of the custom designed instrument and experimental procedure are found in^22^,^23^. In brief, the OT setup was assembled around an inverted microscope (Olympus IX71, Olympus) with a high numerical aperture oil immersion objective (model: UplanFl 100X N.A. = 1.35; Olympus). A continuous Nd:YVO laser (model: Millennia IR) that operates at 1064 nm and is run with an output power of 1.0 W is used as a trap laser during the force measurements. A single bacterium or a probe bead (2.5 μm in diameter) can be trapped by the laser and positioned with nm precision via a computer controlled piezo-stage (E-561.3CD, Physiks Instrument). The position of the trapped probe bead was monitored by projecting the refracted beam of a low power fiber-coupled HeNe-laser, that operates at 632.8 nm with a power of a few hundreds of μm at the sample, onto a position sensitive detector (L20 SU9, Sitek Electro Optics). The stability of the setup was optimized as described in ref. 38, where drift as well as noise were quantified by measuring long time series and analyzing the data using the Allan variance method^39^.

A measurement was performed by capturing a bacterium at low laser power and thereafter mounting the bacterium onto the Poly-L-Lysine coated large bead. Subsequently a suspended small bead was trapped and the stiffness of the trap calibrated, with a typical trap stiffness of 200 pN/μm. The bead was brought into proximity of the bacterium to interact with a fimbria. The trapped bead and bacterium was thereafter separated by translating the piezo-stage thus exposing any attached fimbriae to tensile force. A schematic of how a force spectroscopy experiment is performed is shown in Fig. S3.

### Statistical and computational methods

The mean extension length of fimbriae in the presence and absence of antibodies were statistically compared using an unpaired student t-test. The difference in the length of fimbriae in the presence of antibodies (mean length = 2.9 μm, SD = 0.9 μm) and in the absence of antibodies (mean length = 5.6 μm, SD = 2.2 μm) was significant, with t (30) = −4.33, and a P value of 14.9 × 10^−4^.

The simulated force-extension response of a fimbria fitted to experimental data was performed on a PC using an Intel® Core i7 processor 2.8 GHz and with 16 GB of RAM. The simulation procedure was a slightly modified first-order Markov Chain Monte Carlo (MCMC) Metropolis algorithm as described in^29^. The values of the parameters that minimized the discrepancy between the simulations and measurements provided the best fit.

## Additional Information

**How to cite this article**: Singh, B. *et al.* Antibody-mediated disruption of the mechanics of CS20 fimbriae of enterotoxigenic *Escherichia coli*. *Sci. Rep.* **5**, 13678; doi: 10.1038/srep13678 (2015).

## Supplementary Material

Supplementary Information
