# Supplementary material for: Antibody-mediated disruption of the mechanics of CS20 fimbriae of enterotoxigenic Escherichia coli
Source: Sci Rep. 2015 Sep 28;5:13678. doi: 10.1038/srep13678 (PMC4585931; doi:10.1038/srep13678)
Supplement: Supplementary Information [file srep13678-s1.pdf]

June 8, 2015

Supplementary material:

Antibody-mediated disruption of the mechanics of CS20 fimbriae of enterotoxigenic *Escherichia coli*

Bhupender Singh[a,#], Narges Mortezaei[a,#], Bernt Eric Uhlin[b], Stephen J. Savarino[c,d], Esther Bullitt[e*] and Magnus Andersson[a*]

[a]Department of Physics, Umeå University, SE-901 87 Umeå, Sweden, [b]The Laboratory for Molecular Infection Medicine Sweden (MIMS) and Department of Molecular Biology, Umeå University, SE-901 87 Umeå, Sweden, [c]Enteric Diseases Department, Infectious Diseases Directorate, Naval Medical Research Center, Silver Spring, MD 20910, USA, and [d]Department of Pediatrics, Uniformed Services University of the Health Sciences, Bethesda, MD 20814, USA. [e]Department of Physiology and Biophysics, Boston University School of Medicine, Boston, MA 02118, USA

[#]These authors contributed equally to this work
[*]Corresponding authors
Email: magnus.andersson@physics.umu.se

Running title: Antibodies impede fimbrial elasticity

Key words: IgA, IgG, pili, vaccine, optical tweezers,

June 8, 2015

Force spectroscopy measurements

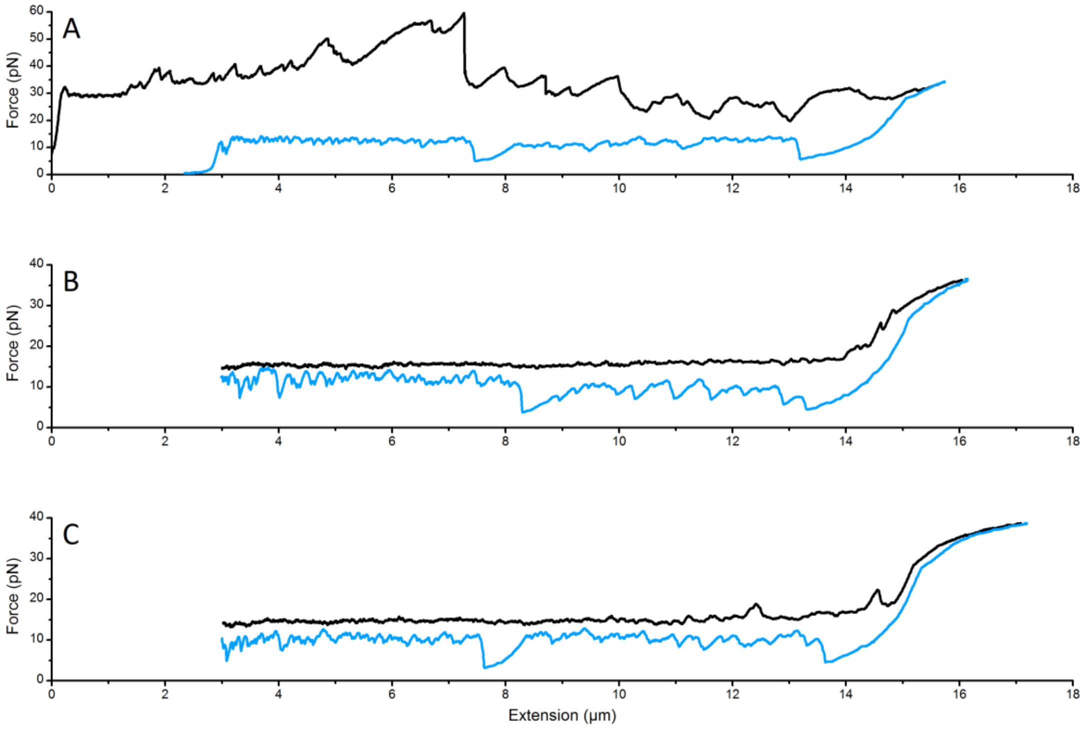

Figure S1. Consecutive force measurements of the CS20 fimbriae in the presence of 0.28 μg/ml of purified anti-CS20 antibodies.

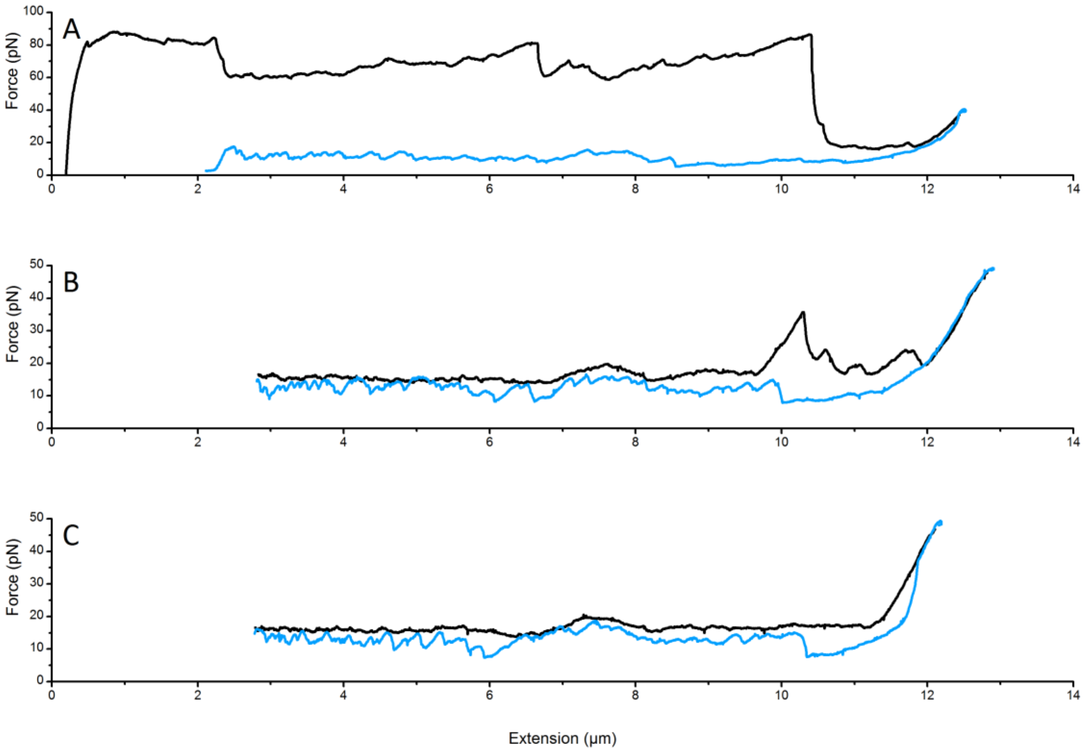

Figure S2. Consecutive force measurements of the CS20 fimbriae in the presence of 0.028 μg/ml of purified anti-CS20 antibodies.

June 8, 2015

Force spectroscopy with optical tweezers

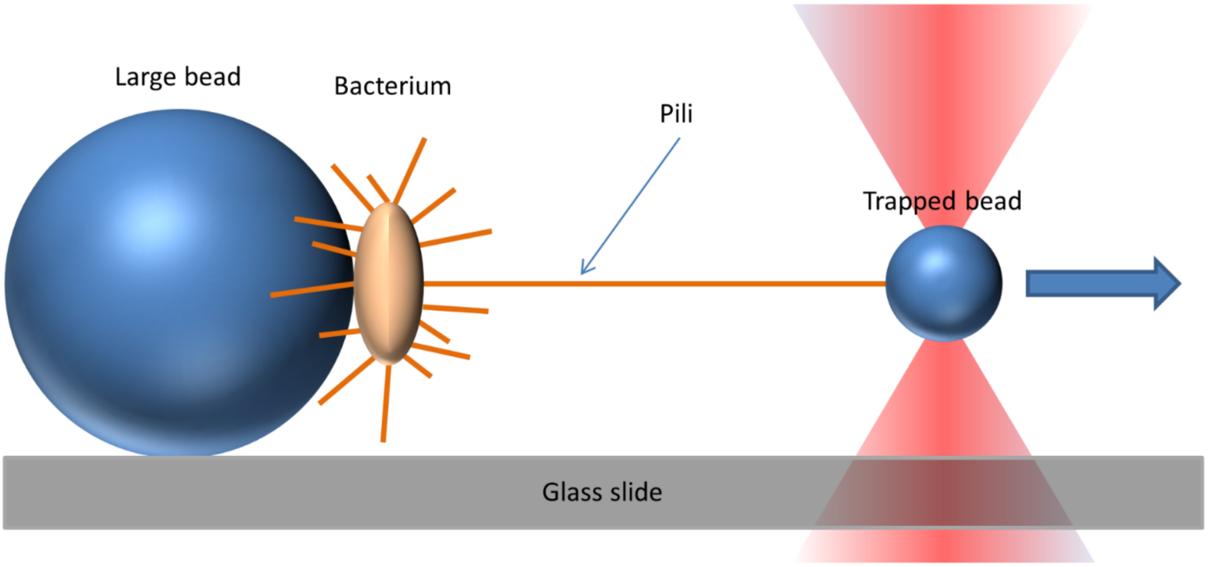

Figure S3. A schematic of how a force spectroscopy experiment on a single fimbria is performed. A 9.5 µm bead is immobilized to a cover slip (Glass slide). The bead is coated with poly-l-lysine which work as an adhesive for a bacterium. Force measurements are performed by trapping a 2.5 µm latex bead and calibrating the trap stiffness using the Brownian motion information. The bead is thereafter attached non-specifically to a fimbria (denoted pili in the illustration). The bacterium-fimbria-bead is thereafter separated by translating a piezo-stage with sub-nm accuracy, thus, the responding force can be assessed at sub-pN force accuracy[1]. The noise and stability of the instrument is prior to measuring a batch of samples analyzed using Allan Variance method[2]. Detailed description of how the experiments are performed can be found in refs.[3,4].

June 8, 2015

Parameter values in the physical model solved using Monte Carlo simulations

Table S1

Model parameter	Value	Description of parameter
ΔV_{AB}	18.0 kT	Energy difference between state A (closed) and B (open).
ΔX_{AB}	5.0 nm	Bond opening length.
ΔX_{AT}	0.4 nm	Distance from state A to energy barrier.
k_A	190 pN/nm	Elastic constant of a subunit in the shaft.

We used the framework of the model presented in ref. [5].

References

1. Andersson, M., Fällman, E., Uhlin, B. E. & Axner, O. Force measuring optical tweezers system for long time measurements of P pili stability. *Proc. SPIE* **6088,** 286–295 (2006).

2. Andersson, M., Czerwinski, F. & Oddershede, L. B. Optimizing active and passive calibration of optical tweezers. *J. Opt.* **13,** 044020 (2011).

3. Axner, O. *et al.* in *Bact. Adhes.* (Linke, D. & Goldman, A.) 301–313 (Springer Verlag, 2011). doi:10.1007/978-94-007-0940-9_19

4. Axner, O. *et al.* in *Springer Ser. Chem. Phys. single Mol. Spectrosc. Chem. Phys. Biol.* (Gräslund, A., Rigler, R. & Widengren, J.) **96,** 337–362 (Springer Berlin Heidelberg, 2010).

5. Björnham, O., Axner, O. & Andersson, M. Modeling of the elongation and retraction of Escherichia coli P pili under strain by Monte Carlo simulations. *Eur. Biophys. J.* **37,** 381–91 (2008).
